# Intravascular ultrasound-derived virtual fractional flow reserve in the superficial femoral artery

**DOI:** 10.1186/s42155-024-00513-y

**Published:** 2024-12-27

**Authors:** Takenobu Shimada, Yoshihiro Iwasaki, Atsushi Funatsu, Tomoko Kobayashi, Shigeru Nakamura, Daiju Fukuda

**Affiliations:** 1 Department of Cardiovascular Medicine, Osaka Metropolitan University Graduate School of Medicine, 1-4-3 Asahimachi Abenoku, Osaka, 545-8585 Japan; 2 Cardiovascular Center, Kyoto Katsura Hospital, Kyoto, Japan

**Keywords:** Physiology, FFR, Functional ischemia, Lumen area, Fluid dynamics, IVUS

## Abstract

**Background:**

Fractional flow reserve (FFR) can be estimated by analysis of intravascular imaging in a coronary artery; however, there are no data for estimated FFR in an extremity artery. The aim of this concept-generating study was to determine whether it is possible to estimate the value of peripheral FFR (PFFR) by intravascular ultrasound (IVUS) analysis also in femoropopliteal artery lesions.

**Methods:**

Between April 2022 and February 2023, PFFR was measured before endovascular therapy in 31 stenotic femoropopliteal artery lesions. High-definition IVUS measurement was simultaneously performed in automatic pullback mode in 6 of those 31 lesions. IVUS-derived PFFR was calculated by an algorithm based on fluid dynamics as the following equation: ΔP = FV + SV^2^. F is the coefficient of pressure loss because of viscous friction (Poiseuille’s equation) and S is the coefficient of local pressure loss because of flow separation (Bernoulli’s equation). The values of F and S were calculated by analysis of IVUS. V is velocity and the value of V was obtained from previously reported data in a duplex ultrasound study. The mean pressure was assumed to be 80 mmHg, and IVUS-derived PFFR was calculated by the following equation: IVUS-derived PFFR = (80 – ΔP) / 80.

**Results:**

The values of IVUS-derived PFFR and actual PFFR were similar: 0.73 and 0.72, 0.87 and 0.92, 0.90 and 0.92, 0.66 and 0.73, and 0.79 and 0.72, respectively. In one case in which run-off of the below-the-knee artery was poor, PFFR (0.91) was higher than the IVUS-derived PFFR (0.73).

**Conclusion:**

Virtual PFFR in the superficial femoral artery can be estimated from IVUS analysis.

## Introduction

Fractional flow reserve (FFR) is an intravascular pressure ratio proximal and distal to a diseased lesion under a maximum hyperemia condition, by which the severity of the functional ischemia of the perfused area can be precisely estimated. In coronary intervention, calculation of virtual FFR, by which the actual FFR value can be estimated from any imaging modality, has become possible [1]. Recently, peripheral FFR (PFFR) has also been reported to be associated with clinical outcomes and imaging findings [2–4]; however, there are no data for estimated PFFR in an extremity artery. The aim of this concept-generating study was to determine whether it is possible to estimate the value of PFFR by intravascular ultrasound (IVUS) analysis also in femoropopliteal artery lesions.

## Concept of IVUS-derived PFFR

**Fig. 1:**
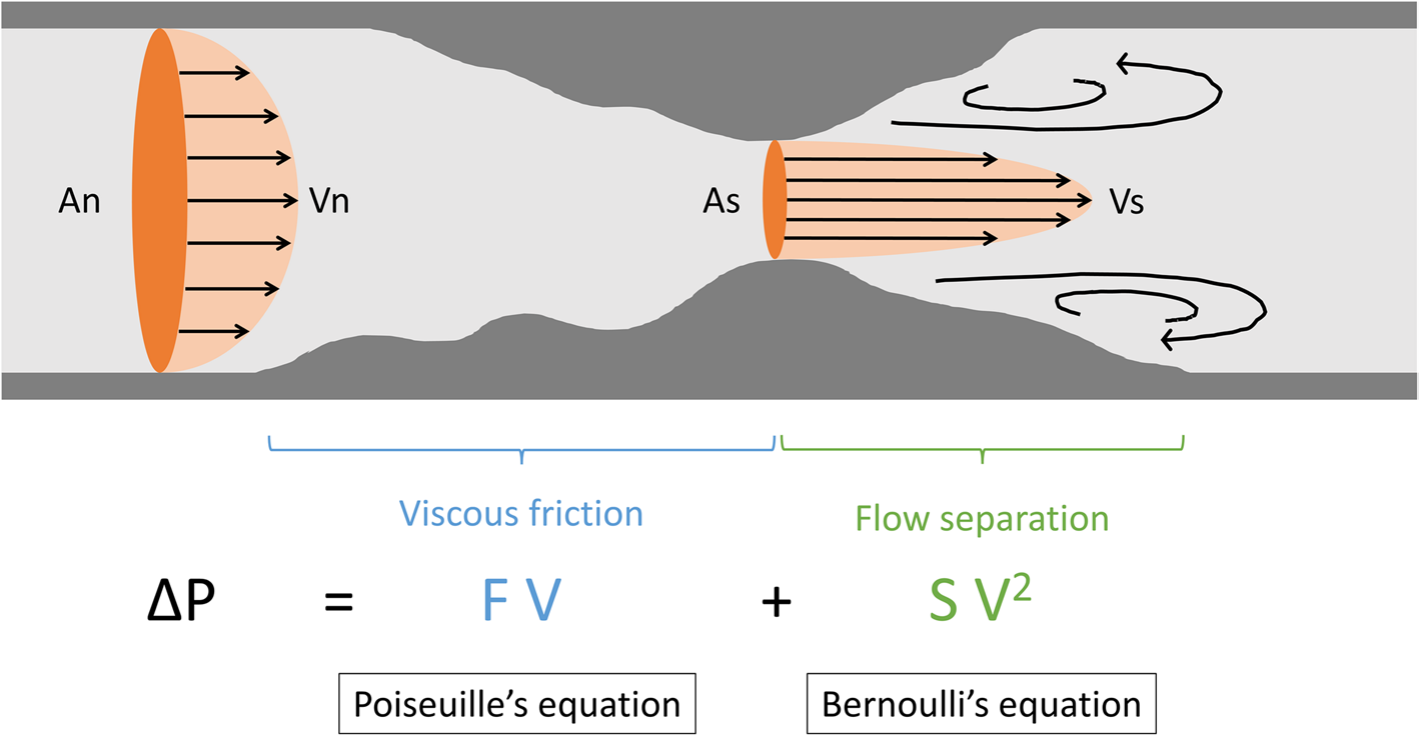
Outline of pressure loss in the stenotic model by fluid dynamics. F is the coefficient of pressure loss because of viscous friction (Poiseuille’s equation), S is the coefficient of local pressure loss because of flow separation (Bernoulli’s equation), and V is flow velocity. F and S were calculated using the following equations: $\:F=\frac{8\pi\:\mu\:L}{As}\frac{An}{As}$ , $\:S=\frac{\rho\:}{2}{\left(\frac{An}{As}-1\right)}^{2}$. F is calculated as the sum of each IVUS slice, and S is calculated through the whole lesion. The value of µis absolute blood viscosity, set as 4.0 × 10 −3 . L is stenosis length, equal to each slice thickness of IVUS images. An is the cross-sectional area of a normal artery (reference lumen area), As is the cross-sectional area of the stenosis segment, and ρ is blood density(1,050 kg/m 3 ). Abbreviations: An, cross-sectional lumen area of the normal segment; As, cross-sectional lumen area of the stenosis segment; Vn, flow velocity at the normal segment; Vs, flow velocity at the stenosis segment; V, flow velocity; ∆P, pressure loss; L, lesion length; IVUS, intravascular ultrasound.

The algorithm for virtual PFFR was based on an algorithm established and validated in a coronary artery model as the following equation: ΔP = FV + SV^2^ [1]. F is the coefficient of pressure loss because of viscous friction (Poiseuille’s equation), S is the coefficient of local pressure loss because of flow separation (Bernoulli’s equation), and V is the flow velocity (Fig. 1). F and S were calculated using the following equations: $\:F=\frac{8\pi\:\mu\:L}{As}\frac{An}{As}$, $\:S=\frac{\rho\:}{2}{\left(\frac{An}{As}-1\right)}^{2}$. F is calculated as the sum of each IVUS slice, and S is calculated through the whole lesion. An is the cross-sectional area of a normal artery (reference lumen area), As is the cross-sectional area of the stenosis segment. The value of µis absolute blood viscosity, set as 4.0 × 10^−3^, L is stenosis length, equal to each slice thickness of IVUS images, and ρ is blood density (1,050 kg/m^3^). Thus, the values of F and S can be calculated by IVUS analysis. V is the flow velocity, and the value of V in a normal superficial femoral artery (SFA) was determined to be 13.4 cm/s in a previous study in which the time-averaged mean velocity (TAMV) was examined by duplex ultrasound [5]. Furthermore, stenotic flow reserve (SFR), which is used for estimating the blood flow increase in a stenotic model under a hyperemia condition [6], was calculated by assuming a mean arterial pressure of 80 mmHg. A previous study showed that the magnitude of increase in blood flow was 5-times higher in a normal SFA under a maximum hyperemia condition [7]. Patient-specific SFR was calculated by the following equation: 80-[F×13.4×SFR+S×(13.4×SFR)^2^]=10+[(100 − 10)/5.0]×SFR [6]. Finally, by assuming the mean pressure was 80 mmHg, virtual PFFR was calculated as follows: IVUS-derived PFFR=(80-ΔP)/80.

### Case series

Between April 2022 and February 2023, actual PFFR was measured using a pressure microcatheter (NAVVUSII; ACIST Medical Systems, MN, USA) before endovascular therapy in 31 stenotic femoropopliteal artery lesions at Kyoto Katsura Hospital. Equalization between the arterial pressure and sensor pressure was performed just outside the tip of the guiding sheath in the CFA. After passing through the target lesion, the tip of the measurement device was placed in the distal segment of the popliteal artery. PFFR was measured after vasodilation with the administration of 30 mg of papaverine through the guiding sheath [7].

High-definition IVUS measurement was simultaneously performed in automatic pullback mode in 6 of those 31 lesions. We calculated virtual PFFR from post-hoc IVUS analysis using an algorithm validated in coronary arteries. Quantitative IVUS measurements were performed using the QIvus 3.1 imaging system (MEDIS Medical Imaging System, Leiden, The Netherlands), and the lumen area was traced automatically and corrected manually.

**Fig. 2:**
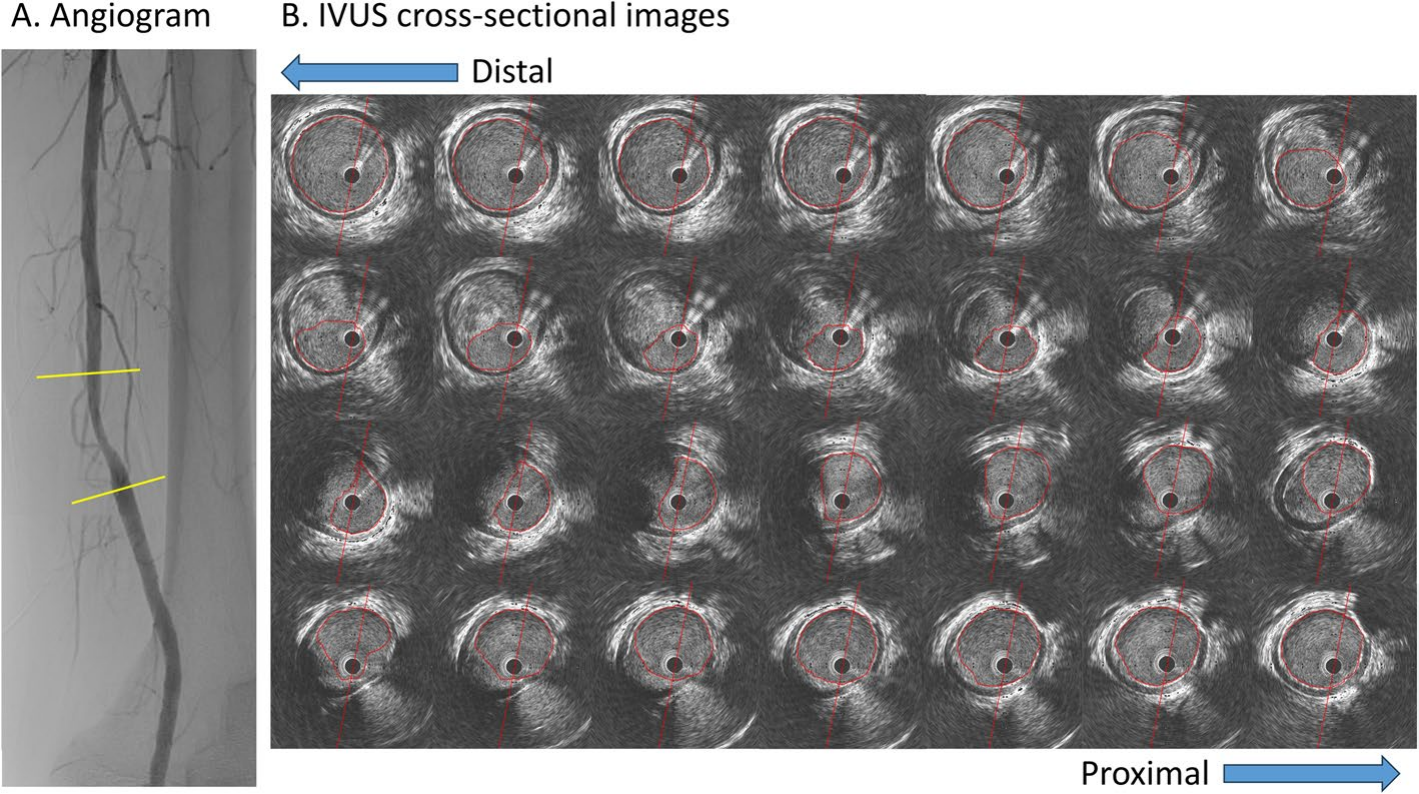
Representative case Luminal surfaces of all cross-sectional IVUS images were traced automatically and corrected manually through the entire lesion. The analysis was performed at 0.167-mm intervals. In this case, a total of 278 slices were analyzed, and 1 out of every 10 images are presented in this figure. Abbreviations: IVUS, intravascular ultrasound.

Table 1 shows the baseline and angiographical characteristics of the patients. A representative case is shown in Fig. 2. In this case, IVUS-derived PFFR and actual PFFR were similar (0.90 and 0.92, respectively).

**Table 1:** Clinical and angiographic characteristics of the target limbs

	Total *n* = 6
Age, y	77 (72–81.25)
Male, n (%)	5 (83.3)
BMI, kg/m^2^	22.4 (18.2–26.5)
Diabetes mellitus, n (%)	2 (33.3)
Hyperlipidemia, n (%)	4 (66.7)
Hypertension, n (%)	6 (100.0)
Smoking history, n (%)	5 (83.3)
Hemodialysis, n (%)	0 (0.0)
eGFR < 45 mL/min/1.73m^2^, n (%)	0 (0.0)
Rutherford class	
1 / 2 / 3	0 (0.0) / 3 (50.0) / 3 (50.0)
/ 4 / 5 / 6	/0 (0.0) / 0 (0.0) / 0 (0.0)
Angiographic analysis	
Reference vessel diameter, mm	4.67 (3.92–5.23)
pre MLD, mm	2.28 (0.70–2.94)
% diameter stenosis, %	49.49 (41.29–82.44)
Lesion length, mm	31.56 (19.53–94.49)

Values are medians (interquartile ranges) and are expressed as numbers (%)

*Abbreviations:* *BMI* body mass index, *eGFR* estimated glomerular filtration rate, *MLD* minimum lumen diameter, *DS* diameter stenosis

Table 2 shows the details of IVUS-derived PFFR and actual PFFR. Although the sample size was small, there was a relatively good correlation between IVUS-derived PFFR and actual PFFR as indicated by Spearman’s rank correlation coefficient of 0.64. By checking the detailed data, the actual PFFR value was higher than the IVUS-derived PFFR in case #4, in which run-off of the below-the-knee (BTK) artery was poor. This is similar to that in a coronary artery in which the actual FFR value is higher than expected from imaging analysis in cases with microvascular resistance.

**Table 2:** Details of IVUS-derived FFR and actual FFR

Case	IVUS-derived PFFR	Actual PFFR	BTK run-off	%DS, %	CLTI	CKD	DM	BMI	ABI
#1	0.73	0.72	3	79.4	N	N	N	18.2	0.53
#2	0.87	0.92	2	44.3	N	N	N	18.2	0.78
#3	0.90	0.92	3	41.7	N	N	Y	20.4	0.91
#4	0.73	0.91	1	54.7	N	N	Y	28.4	0.91
#5	0.66	0.73	2	39.9	N	N	N	25.9	0.89
#6	0.79	0.72	2	91.6	N	N	N	24.3	0.72

*Abbreviations:* *IVUS* intravascular ultrasound, *FFR* fractional flow reserve, *BK* below-the-knee artery, *%DS* percent diameter stenosis, *CLTI* chronic limb-threatening ischemia, *CKD* chronic kidney disease, *DM* diabetes mellitus, *BMI* body mass index, *ABI* ankle-brachial index

## Discussion

The concept-generating analysis in this case study showed that virtual FFR, from which FFR can be estimated by analysis of imaging data, is applicable also for an extremity artery. The physiological dynamics for estimation of PFFR is similar to that of a coronary artery described by the following equation: ΔP = FV + SV^2^ [1].

In the virtual FFR model for a coronary artery, systolic and diastolic velocities are defined separately; however, in this lower extremity artery model, virtual PFFR was calculated on the basis of TAMV without calculating systolic and diastolic velocities separately. The reason of this is that there are no data for the mean velocity validated systole and diastole separately because only peak systolic velocity is evaluated during duplex ultrasound in an extremity artery in daily practice. Further research on the physiological examination is needed to improve the accuracy of virtual PFFR.

In a case with poor run-off of the BTK artery (case #4), the actual PFFR value was higher than the IVUS-derived PFFR. This is similar to that in a coronary artery in which the actual FFR value is higher than expected due to insufficient microvascular vasodilation in cases with microvascular resistance [8]. The microvasculature for the SFA might be the BTK arteries in an extremity artery.

This study was a retrospective cross-sectional study with a small sample size, and our results should be validated in a study with a larger sample size. The patients in this study were those who required EVT, and PFFR measurements before EVT were analyzed; however, more accurate results might be obtained by including lesions that do not require treatment.

In this case study, we evaluated the applicability of IVUS-derived PFFR; however, virtual PFFR by other imaging modalities such as CT or optical coherence tomography (OCT) / optical frequency domain imaging (OFDI) would be desired in the future. CT is a noninvasive imaging modality that is commonly used in daily practice, and estimation of distal perfusion by CT would be beneficial. OCT / OFDI has a higher automatic lumen traceability than that of IVUS, and verification of virtual PFFR by analysis of OCT / OFDI would be desirable. It has been reported that PFFR was associated with long-term patency after EVT [3], and accurate virtual PFFR estimated from imaging analysis may also enable prediction of long-term patency.

## Conclusion

PFFR in the SFA may be estimated from IVUS analysis. Estimation of physiological dynamics from an imaging modality can be performed similarly to that for a coronary artery.

## Data Availability

The datasets used and/or analyzed during the current study are available from the corresponding author on reasonable request.

## Abbreviations

PFFR: peripheral fractional flow reserve
IVUS: intravascular ultrasound
EVT: endovascular therapy
SFA: superficial femoral artery
SFR: stenotic flow reserve
